# Structural Evidence of Interanionic Hydrogen Bonding in Phosphoric Acid Solutions

**DOI:** 10.1021/jacs.5c12699

**Published:** 2025-11-24

**Authors:** Pyeongeun Kim, Richard Kang, Kevin Carter-Fenk, Kevin R. Wilson, Martin Head-Gordon, Musahid Ahmed

**Affiliations:** † Chemical Sciences Division, 1666 Lawrence Berkeley National Laboratory, Berkeley, California 94720, United States; ‡ Department of Chemistry, 1438 University of California, Berkeley, California 94720, United States; § Department of Chemistry, 6614 University of Pittsburgh, Pittsburgh, Pennsylvania 15218, United States

## Abstract

Interanionic hydrogen bonding (IAHB) is a noncovalent interaction between like-charged ions that challenges conventional electrostatic understanding. This study provides direct structural evidence of IAHB in concentrated aqueous phosphoric acid (PA) solutions, which exhibit >60% dissociation under these conditions. Oxygen K-edge X-ray absorption fine structure spectroscopy, combined with electron affinity time-dependent density functional theory calculations, reveals the formation of stable, cyclic phosphate-phosphate IAHB dimers at PA concentrations ≥7 M. Extended X-ray absorption fine structure data show distinct long-range ordering consistent with these dimers, and near-edge X-ray absorption fine structure spectra confirm a concentration-dependent transition from monomeric to dimeric species. Energy decomposition analysis through density functional theory shows that the formation of solution-phase IAHB is energetically favored and is attributed to polarization of and the charge transfer between the two fragments driven by the surrounding solvent molecules, in addition to the permanent electrostatics. These findings offers crucial structural insights into the H-bonded networks in concentrated PA, highlighting the critical role of solvent in facilitating anion–anion association.

## Graphical abstract

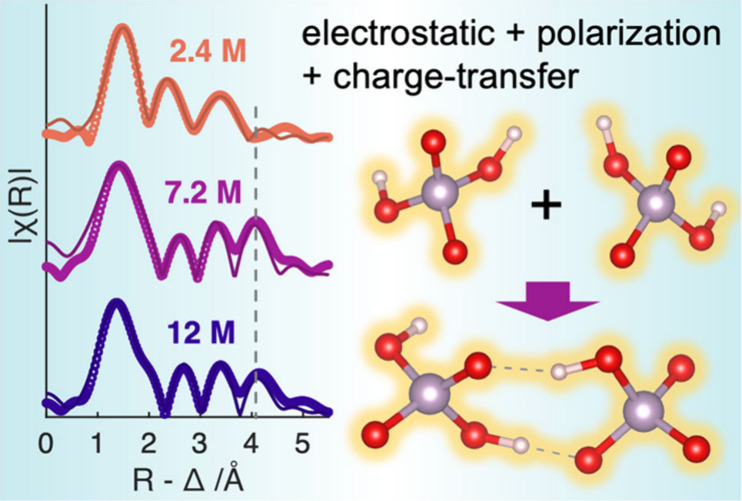

## Introduction

Hydrogen bonding (H-bonding) is a fundamental noncovalent interaction that governs structure and dynamics across molecular systems. Molecular assembly and associated functions are dictated by H-bonding in various important systems, including water, DNA, proteins, ionic liquids, and new types of porous materials such as H-bonded organic frameworks (HOFs).^1–6^ While energetics and structures of conventional H-bonds between neutral–neutral and neutral–charged species have been extensively studied, there is much that remains to be understood at the molecular level of H-bonding between like-charged ions.^7–9^ The interactions between like-charged ions, and strategies to tune them, have broad implications for both the fundamental understanding of noncovalent forces and the design of new material scaffolds with tunable properties.^5,6,10,11^ The interplay of Coulombic forces, selective hydrogen bonding, and dispersion interactions offers a foundation for hypothesis-driven design principles with applications in synthesis, separations, biology, and electrochemistry.

Despite the apparent contradiction between Coulombic repulsion and directional association, theoretical and crystallographic studies have reported that H-bonding between like-charged ions can be formed in specific chemical environments.^11–14^ This phenomenon has been described as “antielectrostatic hydrogen bonding” (AEHB), though this terminology remains contentious. Alkorta et al. recently raised concerns regarding the potential for misleading interpretation and use, and suggested more physically accurate terminology, such as interanionic or intercationic H-bonding (IAHB or ICAB).^15^

Phosphoric acid (PA) and phosphates offer a particularly interesting system to explore IAHB.^16^ As central components of many biological systems (nucleic acids, membranes, cofactors, etc.), phosphate groups play essential structure-defining roles in molecular biology, often engaging in metal cation-assisted IAHB.^17,18^ Recent studies have reported phosphate-phosphate IAHB identified through nuclear magnetic resonance (NMR) and X-ray crystallography.^11,19^ The reported IAHB in crystalline and solution phases are stabilized by either positive counterions or supramolecular anion receptors, respectively, thus compensating for electrostatic repulsion.^20,21^ While ^1^H NMR is commonly used to probe IAHB in solution, it is often limited by proton–deuterium exchange and line broadening due to chemical exchange, complicating molecular-scale structural analysis.^11^ IAHB has also been postulated as the source of frequency shifts in vibrational fingerprints of PA solutions,^22^ though the hypothesis remains debated due to a lack of structural evidence. Theoretical studies have also focused on H-bonding between like-charged ions and predicted the formation of stable phosphate IAHB in a polarizable medium.^23–25^ However, experimental evidence of IAHB structure in an aqueous environment without the aid of anion receptors remains lacking.

Interestingly, H-bonding networks of PA solutions have been extensively studied since the early 60s, owing to their excellent proton conductivity. Studies suggest that the Grotthuss mechanism, the rapid proton transfer process through the successive breaking and reforming of H-bonds in an intermolecular network,^26,27^ as the cause of exceptional proton conductivity of concentrated PA aqueous solutions.^28–30^ Although PA is known as a weak acid (p*K*_1_ = 2.14), its dissociation into H_2_PO_4_^–^ increases with concentration, reaching ∼60% at 10 M.^31,32^ For Grotthuss-type proton transfer via extended H-bond network to occur in such highly concentrated PA solutions with >60% of phosphate anions (H_2_PO_4_^–^), the possible anion–anion structure must be considered, raising the question of the concentration regime at which these interactions become favorable. The evolution of H-bonding network and associated conductivity versus concentration has direct implications for energy storage technologies, particularly in PA fuel cells where water content near the electrode can vary significantly.^33,34^

Beyond PA, studies of other oxoacids have provided related insights into how liquid structures evolve at high concentrations. Voth and co-workers recently proposed that metastable shared-proton complexes form in aqueous sulfuric acid, where ab initio molecular dynamics (AIMD) simulations indicated contact ion pairs between HSO^4–^ and SO_4_^2–^ at ∼0.5 M.^35^ In collaboration with Fayer and co-workers, they also used ultrafast optical Kerr spectroscopy to probe proton-hopping lifetimes in sulfuric acid solutions up to 18.3 M, focusing on conductivity and local H-bond dynamics.^36^ Earlier, Hemminger and co-workers applied liquid-jet X-ray photoelectron spectroscopy to sulfuric acid and observed concentration-dependent speciation changes around 5–7 M, possibly due to restructuring of the solvation environment.^37^ Similar trends were reported for nitric acid, where XPS and MD revealed that concentrations >4 M lead to more structured solvation shells, driven by overlap of HNO_3_ and NO_3_^–^ solvent shells and reduced water mediation between ions.^38^ Collectively, these studies demonstrate that concentrated acid solutions undergo substantial reorganization of their hydrogen-bonding networks and solvation structures.

Here, we provide direct structural evidence of phosphate–phosphate IAHB in PA solutions by using aerosol-based oxygen K-edge X-ray absorption fine structure spectroscopy (XAFS) and density functional theory (DFT) calculations. Extended X-ray absorption fine structure (EXAFS) and structural modeling provide angstrom-scale structural evidence of the IAHB dimer formation in ≥7 M PA solutions. Near-edge X-ray absorption fine structure (NEXAFS) spectra exhibit concentration-dependent changes, consistent with a transition from monomeric to dimeric configurations supported by electron-affinity time-dependent density functional theory (EA-TDDFT) calculations. Lastly, the conditions that favor IAHB dimer formation are analyzed via energy decomposition analysis (EDA) based on density functional theory (DFT) calculations. Near-edge X-ray absorption fine structure (NEXAFS) spectra exhibit concentration-dependent changes, consistent with a transition from monomeric to dimeric configurations supported by electron-affinity time-dependent density functional theory (EA-TDDFT) calculations. Lastly, the conditions that favor IAHB dimer formation are analyzed via energy decomposition analysis (EDA) based on DFT calculations.

## Results and Discussion

To identify experimental evidence for the structure of the phosphate IAHB dimer as shown in Figure 1a, a set of PA solutions was analyzed by oxygen K-edge EXAFS. This technique provides oxygen-specific, local structures in solution in angstrom-scale resolution.^39,40^ A schematic of the experimental setup is shown in Figure 1b, with further details provided in the Supporting Information (SI). In brief, PA solutions are aerosolized by an atomizer, then delivered into the X-ray interaction region via an aerodynamic lens system (ADL).^41,42^ PA solutions were prepared in the range of 2–10 M. Upon introduction into the instrument, aerosol droplets undergo partial evaporation, leading to an estimated increase in solute concentration of up to ∼20%.^43^ Unless otherwise noted, all concentrations reported in this work refer to these post-evaporation values. Aerosol delivery was chosen over a liquid jet because it is more robust at high ionic strength and viscosity, avoids jet-collection hardware, and allows for straightforward electron detection for XAFS. X-ray photoelectrons generated from O atoms in aerosols are focused into an imaging detector consist of a microchannel plate (MCP) and a phosphor screen (Figure S1). The total number of photoelectrons is measured by a photomultiplier tube (PMT). The X-ray absorption intensity is therefore, PMT signal normalized by flux of the X-ray beam measured by a photodiode (*I*_XA_ = *I*_PMT_/*I*_PD_). For EXAFS measurements, *I*_XA_ is measured over *h*ν = 520–750 eV (Figure S2), while NEXAFS measurements cover the 532–552 eV range. Absolute photon energy calibration of the X-ray monochromator was achieved by measuring well-characterized 1s to π* transitions of gas phase N_2_ (401.0 eV) and O_2_ (530.8 eV).^44,45^ XAFS capture time-averaged snapshots of local solvation environments, largely unaffected by solvent dynamics or proton mobility on longer time scales (hundreds of femtoseconds), due to the ultrafast time scale of X-ray core-to-valence excitation (few femtoseconds).^46–49^ NEXAFS spectra of aerosolized solutions show the aerosols remain in the liquid phase at the X-ray interaction region although the fast cooling upon entering the instrument through the ADL leads to an estimated temperature of ∼200 K (vide infra).^43^ Although evaporative cooling lowers droplet temperatures, it primarily extends structural lifetimes,^42,50,51^ and the observed phosphate–phosphate IAHB reflects intrinsic interactions that can persist at room temperature.

**Figure 1:**
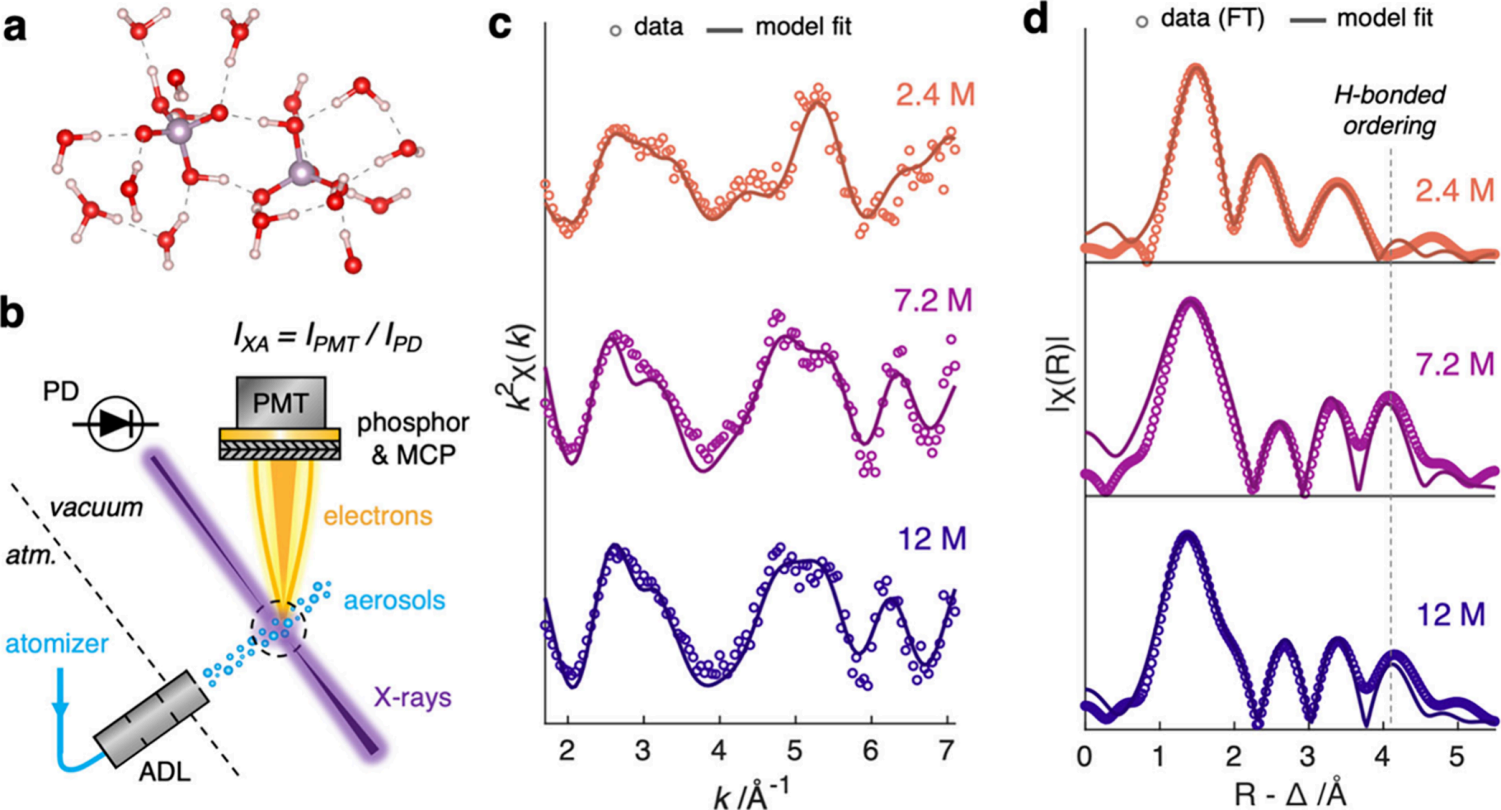
Sample dimer configuration and aerosol XAFS measurements. (a) A snapshot of phosphate–phosphate cyclic H-bonded dimer predicted by DFT calculations. (b) Simplified experimental schematics of an aerosol-based X-ray spectrometer at the Advanced Light Source beamline 9.0.1. ADL is an acronym for aerodynamic lens system. The value of the X-ray absorption intensity (*I*_XA_) is obtained by normalizing the photoelectron signal measured by a photomultiplier tube (*I*_PMT_) with the X-ray intensity measured by a photodiode (*I*_PD_). (c) *k* ^2^-weighted and (d) Fourier-transformed (FT) EXAFS oscillations from 2.4, 7.2, and 12 M [PA] aerosols. Sets of experimental (circles) and modeled (solid lines) results are shown.

### Structural Analysis using EXAFS

The resulting oxygen K-edge EXAFS spectra reveal clear concentration-dependent structural evolution, as shown in Figure 1c,d. Concentrated PA solutions (7.2 and 12 M) exhibit significant long-range ordering that is absent in lower concentration (2.4 M). Figure 1c presents the *k* ^2^-weighted EXAFS oscillations (*k*-space, unit: Å^–1^) for 2.4, 7.2, and 12 M PA solutions. The corresponding moduli of Fourier-transformed spectra (R-space, unit: Å), containing information about interatomic distances, are shown in Figure 1d. Note that the Å scale in the R-space EXAFS is systematically shorter by 0.3–0.5 Å compared to the true interatomic distance due to the phase shift during scattering events, thus noted as R-Δ in Figure 1d. In *k*-space, the 7.2 and 12 M exhibit similar structure, with more pronounced high-frequency oscillation that can be seen at 5–7 Å^–1^ which is suppressed in the 2.4 M as a result of destructive interference (Figure 1c and Figure S3). These high-frequency oscillations manifest as longer-range interactions at ∼4 Å in the R-space (Figure 1d), suggesting the emergence of intermolecular ordering in 7.2 and 12 M PA is likely induced by H-bonding.

To obtain quantitative structural information and confirm the molecular nature of this ordering, ab initio molecular dynamics (AIMD) simulations of hydrated PA monomer and dimer are performed, and the 40 ps trajectories are used as baselines to fit FEFF8 structural model to the EXAFS data (Figure S4).^49,52^ By varying constraints on the phase-shift energy (*E*_0_), degeneracy (*N*), and amplitude factor (*S*_0_^2^), three physically reasonable fittings are converged for each PA concentration. All model fitting results are shown in Figure S5, and the values of all fitting parameters (*r*, *N*, σ^2^, *S*_0_^2^, and *E*_0_) and the goodness of fit (R-factor) are tabulated in Tables S1–S3. The best-fitting results shown in Figure 1 are determined based on R-factor value and the degree of phase-shift (*E*_0_). The parameter *r* (i.e., scattering path length or true interatomic distance) represents half of the total path length traversed by photoelectrons between the excited O atom and neighboring P or O atoms (i.e., scattering bodies). Schematic illustrations of the modeled structures for 2.4 and 7.2 M PA and deconvoluted scattering paths of hydrated monomer and dimer models are provided in Figure 2.

**Figure 2:**
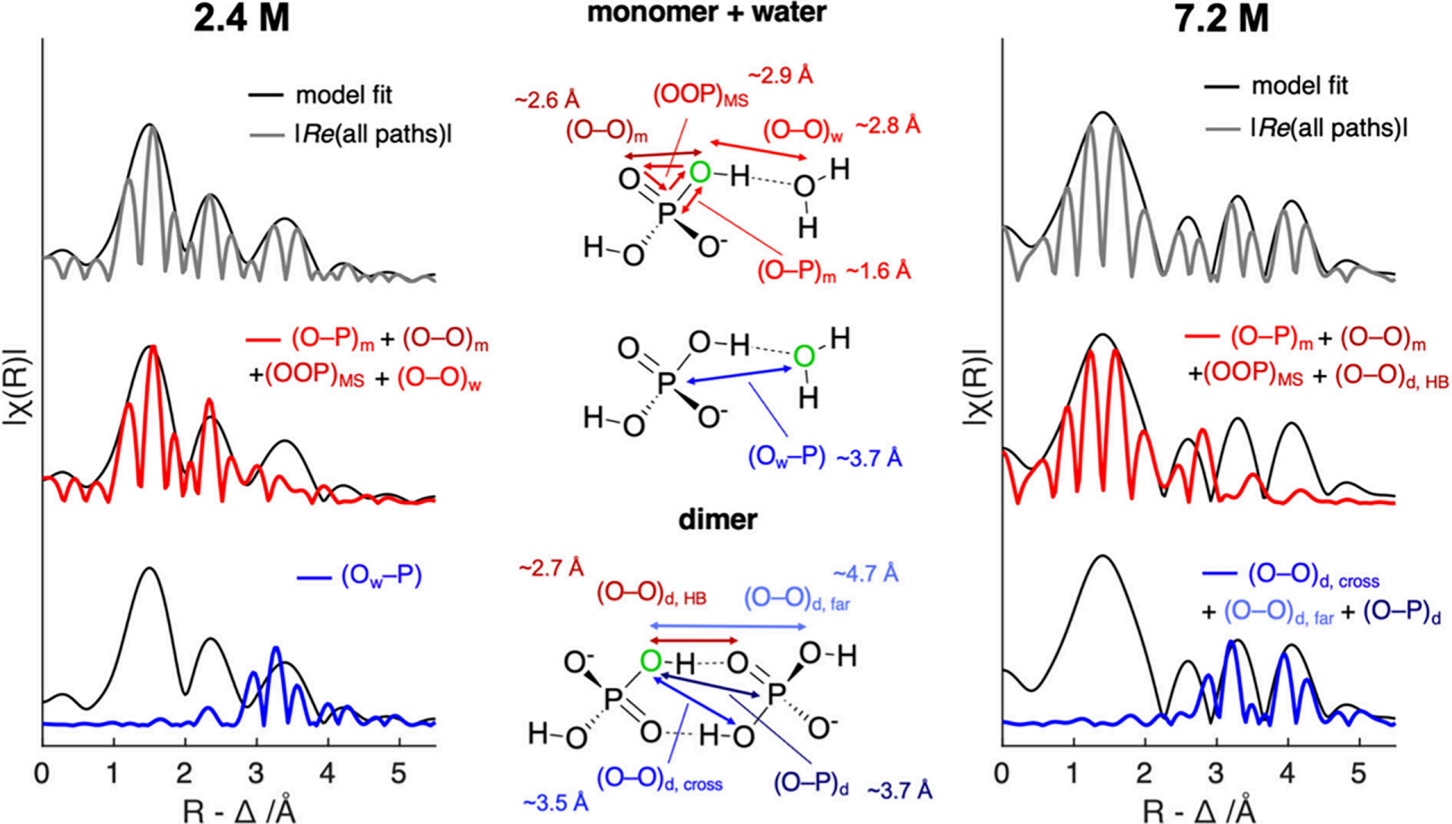
Structural model used for EXAFS fitting. (center) Hydrated monomer and cyclic dimer model of PA. X-ray absorbing O atom is colored in green. Note that water molecules in the hydration shell but one in the monomer model are omitted from the scheme for simplicity. (left) Modulus of best-fit EXAFS model of 2.4 M PA (black lines) overlaid with the sum of real part of the FT scattering paths (gray line). Red line shows the sum of shorter-range (<3 Å) intramolecular and H-bond scattering paths. Blue line shows the major contribution of >3 Å features come from the (O_w_–P) scattering path. (right) For 7.2 M PA, longer-range contributions (blue line) originate from (O–O)_d, far_, (O–O)_d, cross_, and (O–P)_d_ scattering paths of the cyclic H-bonded dimer. EXAFS of 12 M PA exhibit features similar to those of 7.2 M (Figure S6).

As shown in Figure 2, the 2.4 M EXAFS structure matches well with the monomer model, where a phosphate is fully solvated by water.^53,54^ The ∼3.5 Å R-space peak in the 2.4 M data originates from the water oxygen–phosphorus scattering path (O_w_–P) within the PA–water interaction, as oxygen in water molecule can also generate photoelectrons via X-ray absorption (Figure 2, center). The absence of dimeric structures in 2.4 M solution is evident by the lack of significant R-space EXAFS features beyond 4 Å in contrast to the 7.2 and 12 M data. Therefore, the EXAFS data and model fitting suggest the intermolecular ordering in 2.4 M solution only exists within the first layer of phosphate–water solvation shell structures up to P–O···O distance of ∼3.7 Å ((O_w_–P) in Table S1), which value is in line with AIMD result by Kundu et al.^49^ It should be noted that the contribution from the H-bonded water–water (O–H···O) scattering path is assumed to be included in the (O–O)_w_ path (*r* = 2.75 Å) to keep a reasonable number of unknown parameters in the model.

In contrast, at 7.2 and 12 M, both EXAFS structures are best reproduced by the phosphate dimer model, as shown in Figure 2 and Figure S6. Long-range intermolecular (O–P)_d_, (O–O)_d, cross_, and (O–O)_d, far_ scattering paths contribute to R-space peaks beyond 3 Å (Figure 2, bottom right). This indicates the formation of stable, cyclic H-bonding networks between phosphates and PA at higher concentrations. The proposed dimeric structure shown in Figure 2 is consistent with an early wide-angle X-ray scattering results of 85 wt % PA solution (57.8 molal) by Wertz and Cook.^55^ Notably, the onset of dimer formation in [PA] ≥ 7 M parallels X-ray studies of other oxoacids, where restructuring of the hydration environment due to solvation shell overlap has been observed around 4–7 M for nitric and sulfuric acids.^37,38^

All R-space EXAFS spectra display a strong peak at ∼1.5 Å and a weaker one at ∼2.3 Å. The former mainly corresponds to the intramolecular (O–P)_m_ scattering path, and its value obtained from the fitting (∼1.6 Å) is in excellent agreement with literature (Figure 1d).^53,55,56^ The latter arise from multiple overlapping contributions, including multiple O–O–P scattering ((OOP)_MS_) and both intramolecular and intermolecular O–O paths ((O–O)_m_, (O–O)_w_, and (O–O)_d, HB_) (Figure S7). It is worth noting that the destructive interference of these medium-range paths leads to the apparent dip at ∼2.3 Å in the R-space EXAFS (Figure 1d and Figure S7). For 7.2 and 12 M data, the contribution from water–water scattering is likely included in this 2–3 Å region, although it is difficult to quantify its physical parameters due to the overlap with multiple paths of similar distances ((OOP)_MS_, (O–O)_m_, (O–O)_w_, and (O–O)_d, HB_).

### Evolution of Intermolecular Interactions via NEXAFS

The structural information obtained by EXAFS provides compelling evidence for the formation of a stable IAHB phosphate dimer in ≥7.2 M solutions. While EXAFS offers clear structural evidence for dimer formation, it does not provide detailed insight into the evolving local hydrogen-bonding environment as a function of concentration. To address this, NEXAFS measurements and EA-TDDFT^57^ calculations were performed. NEXAFS offers valuable insight into the evolution of H-bonding by accessing excitations from core orbitals into highly polarizable antibonding orbitals, which can yield important information about the local hydrogen bonding interactions.^58^ Conventional linear-response TDDFT has generally shown significant underestimation of core-hole excitation energies.^59,60^ However, recent theoretical developments have improved its performance on predicting core–hole states,^61,62^ enabling computation of accurate linear and nonlinear spectra of solvated systems.^63–65^ EA-TDDFT used in this work improves upon conventional linear-response TDDFT through a core-hole optimized reference state.^57^ In conjunction with explicit water solvation shells extracted from classical MD, EA-TDDFT has been shown to reproduce key spectral signatures of liquid/solution near-edge spectra.^47^ We therefore used NEXAFS in combination with EA-TDDFT to identify spectral signatures supporting PA dimer formation in solution.

Figure 3a shows the evolution of NEXAFS from [PA] = 0.2–12 M, including spectra of water and ice from the work of Frati et al.^45^ for comparison. In all PA cases, the dominant absorption edge at 538.0 eV indicates that the aerosolized droplets remain as liquid while interacting with the X-ray beam, despite evaporative cooling to an estimated temperature of ∼200 K.^43^ The absence of a pronounced postedge peak compared to the ice spectrum, supports this argument, as the liquid-to-solid phase transition typically results in a sharp spectral feature at higher energies near 542 eV.^66–68^ The spectrum of the 0.2 M solution reflects a system predominantly composed of water, as the oxygen spectral contribution from PA is minimal (<1.5 mol %). Notably, this spectrum lacks the characteristic pre-edge feature of neat liquid water at ∼535 eV, which is generally attributed to broken hydrogen bonds.^69^ This suppression may indicate that the aerosolized droplets at this concentration may exist in a supercooled state with a relatively intact H-bond network, consistent with previous studies reporting stable supercooled phases in submicron-sized water droplets due to finite-size and surface effects, even at temperatures as low as ∼180 K.^70,71^ At [PA] < 6.0 M, two distinct peaks appear in the NEXAFS spectra at 533.9 and 535.9 eV. These peaks are absent in the dilute 0.2 M spectrum, suggesting they originate from the phosphate/PA moieties rather than from water. These two transitions then disappear in [PA] ≥ 7.2 M. This suggests that a structural change in PA, such as dimer formation, is suppressing these two pre-edge transitions.

**Figure 3:**
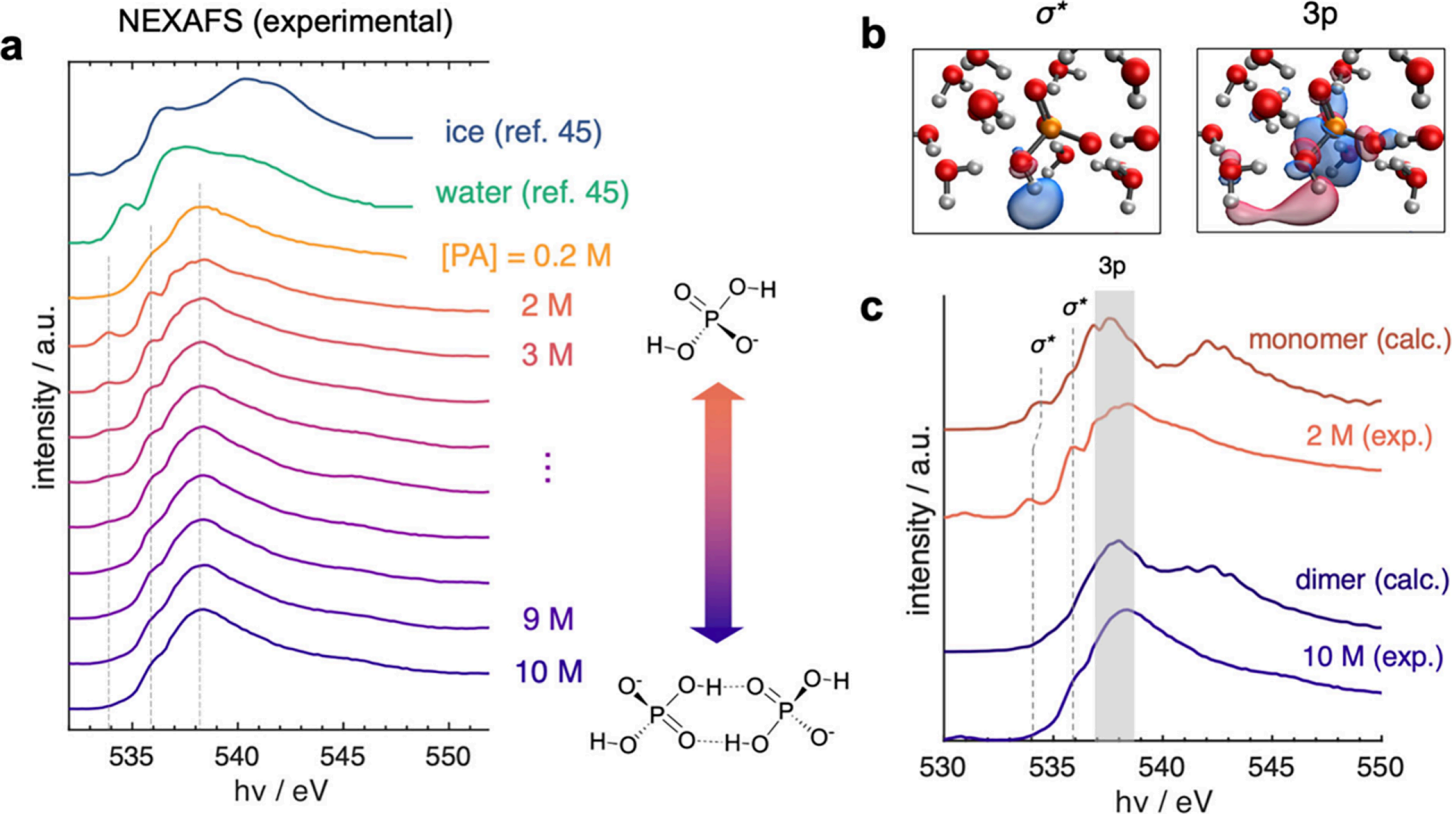
Experimental and statistical data for (EA-TDDFT) NEXAFS. (a) Concentration-dynamic NEXAFS measurements of PA solution with [PA] range of 0.2 to 12 M. For comparison, spectra of pure ice and water adapted from ref 45 are included. Two pre-edge and the main-edge features of PA solutions are highlighted in dashed gray lines. (b) Computed natural transition orbitals (NTO) of the O 1s to OH σ* and the O 1s to 3p transitions for the monomer spectra. The orange, red, and white atoms indicate phosphorus, oxygen, and hydrogen atoms, respectively. The isosurface contains 30% of the electronic density, highlighting the strongest character of the excitations. (c) Experimental NEXAFS spectra (2.4 and 12 M PA) compared against calculated spectra of monomer and dimer models. The good agreement in the near edge suggests that 12 M experimental data are dimer-dominated and the 2.4 M experimental data is monomer-dominated. No empirical shifts are applied to the calculated spectra.

To provide molecular-level explanation for the spectral evolution, the EA-TDDFT calculations under the Tamm–Dancoff approximation were performed on 41 PA monomer and dimer geometries with explicit second solvation shells. These geometries were extracted from two 40 ps AIMD trajectories which sampled monomer and dimer configurations, respectively, and were also used in the EXAFS model analysis (Figure 1a and Figure 2). The first and second explicit shells were determined from computed P–O radial distribution function based on AIMD trajectories (Figure S4). Excitations were restricted to the oxygen K-shells of PA to minimize contributions arising from H_2_O. The calculated monomer spectrum and the experimental 2.4 M spectrum shown in Figure 3c both show characteristic pre-edge transitions.

The two transitions at 533.9 and 535.9 eV were identified as O 1s to OH σ* transitions of O in phosphate by computing natural transition orbitals and their electron density is shown in Figure 3b .^72^ The particle orbital of the O 1s to OH σ* transition is mainly centered on the OH bond. The blue-shifted peak (535.9 eV) is characterized by partial charge transfer to a nearby water (Figure S8a). Both features, however, disappear in the calculated dimer and the experimental 12 M spectrum. Since these two transitions involve the antibonding orbital along the O–H bond of the phosphate, we can postulate that the H-bond formed between two monomers suppresses these transitions. Notably the brightest (i.e., edge) transitions at ∼538 eV for both monomer and dimer spectra are attributed to linear combinations of O 3p orbitals both from phosphate and waters near the excitation center (Figure 3b and Figure S8b, respectively). While these orbitals are spatially diffuse and delocalized across multiple oxygen atoms, they remain primarily centered within the phosphate and are likely less affected by the [PA] change and intermolecular H-bonding structure (Figure 3b). The good agreement in the pre-edge spectra allows us to draw these conclusions; the disparity in the postedge region (540–545 eV) reflects known limitations of EA-TDDFT beyond the ionization threshold.^59^

Complementing EXAFS data, the NEXAFS results provide a clear picture for a concentration-dependent evolution of the local H-bonding structure. At lower concentrations, the spectral features are consistent with monomeric PA–water (and phosphate–water) interactions, while at relatively higher concentrations (≥7 M), the data show evidence for the formation of dimeric PA–PA and phosphate–phosphate structures (Figure 3a). A linear combination analysis of the spectra using 2.4 and 12 M as structural end points reveals a linear increase in dimer population with decreasing H_2_O:PA ratio, as shown in Figure S9. This transition suggests the formation of extended H-bonded network of PA for Grotthuss-type proton transfer becomes more likely in higher [PA], as predicted in MD simulations by Mikalčiu̅tė et al.^73^ Since EXAFS and NEXAFS used in this study are mostly sensitive up to <5 Å first-order dimeric interactions (Figures 2 and 3), the experimental results demonstrate the possible structure of the dimeric unit including IAHB phosphates, which can be the building block of higher-order H-bonded network in concentrated PA solutions critical for proton hopping mechanism.^36^

### Energy Decomposition Analysis of the Anionic Dimer

The combined experimental and theoretical analyses provide support for the formation of IAHB in concentrated PA solutions, even in the absence of macromolecular acceptors and counterions. Naturally, a question arises: Is H-bonding between phosphates strong enough to overcome Coulombic repulsion?^12^ A previous study by Espinosa et al. has directly tackled this question by assessing the potential energy surfaces of various dianoic PA dimers in the gas phase.^13^ The study identified a favorable electric field generated by permanent electrostatics between the H-bond donor and acceptor and concluded that the structure forms a local minimum strong enough to balance out the ionic repulsion. Alkorta et al. have subsequently criticized describing the nature of any hydrogen-bonding as antielectrostatic.^15^ Mo et al. have further utilized a block-localized wave function method and have thus identified the charge-transfer between monomer fragments to be crucial in forming the metastable IAHB dimer in the gas phase.^74^

Here, we revisit this question using a similar energy decomposition analysis^75–79^ based on absolutely localized molecular orbitals (ALMO-EDA)^80–85^ with density functional theory (DFT). While similar to the work by Mo et al.,^74^ we differ here by directly accounting for the role of solvation, both implicitly and explicitly. First, we computed the formation energy of a PA dimer using simple dimer models based on geometries optimized by DFT. While previous studies have examined various conformations of PA dimer,^86^ here we focused on the role of solvation and have chosen a dimer configuration with two H-bonds present. Three systems were optimized to compute the energy of formation (Δ*E*_FORM_) under different conditions: (1) a dimer in the gas phase, (2) a dimer embedded in an implicit polarizable continuum medium (PCM) solvent, and (3) a dimer with an explicit first solvation shell embedded in PCM solvent. Six explicit water molecules were used so that every oxygen atom in the PA dimer formed an explicit hydrogen bond with either the oxygen of another water molecule or the oxygen of the other PA in the dimer. Each of the optimized structures is provided in the SI. Equations 1–3 describe the models we used:

1 $$2{\left[H_{2}{\mathrm{PO}}_{4}\right]}^{-}\;\left(g\right)\to {\left[{\left(H_{2}{\mathrm{PO}}_{4}\right)}_{2}\right]}^{2-}\;\left(g\right)$$

2 $$2{\left[H_{2}{\mathrm{PO}}_{4}\right]}^{-}\;\left(\mathrm{PCM}\right)\to {\left[{\left(H_{2}{\mathrm{PO}}_{4}\right)}_{2}\right]}^{2-}\;\left(\mathrm{PCM}\right)$$

3 $$2{\left[H_{2}{\mathrm{PO}}_{4}{\left(H_{2}O\right)}_{6}\right]}^{-}\;\left(\mathrm{PCM}\right)\to {\left[{\left(H_{2}{\mathrm{PO}}_{4}\right)}_{2}{\left(H_{2}O\right)}_{6}\right]}^{2-}\;\left(\mathrm{PCM}\right)+{\left(H_{2}O\right)}_{6}\;\left(\mathrm{PCM}\right)$$

Table 1 shows the computed formation energies. It is evident that the dimer interaction is energetically disfavored in the gas phase, but changing to polarizable continuum model (PCM) solvation makes the dimer formation favorable. Interestingly, the inclusion of the first explicit solvation shell further stabilizes this reaction by ∼7 kJ/mol. While this is significantly smaller than the strength of a typical water-to-water H-bond, it may suggest that the first solvent shell H-bonding slightly assists in the formation of the dimer. In particular, the loss of some H-bonds between water and PA associated with dimer formation does not carry an energetic penalty.

**Table 1:** Computed H_2_PO_4_^–^ Dimer Formation Energy As a Function of the Environment^a^

formation equation	embedded in PCM	explicit 1st solvation shell	Δ*E*_FORM_ (kJ/mol)
[Disp-formula eq1]	no	no	125.38
[Disp-formula eq2]	yes	no	–62.99
[Disp-formula eq3]	yes	yes	–70.43

a All optimized geometries are listed in the SI.

To further examine the role of the first solvation shell, we performed a set of EDA calculations on our three dimer formation models to decompose the interaction energies of the IAHB dimer. In brief, EDA decomposes the interaction energy Δ*E*_INT_ of a dimer into the frozen interaction of the two fragment orbitals (Δ*E*_FRZ_), polarization/induction of the fragment orbitals (Δ*E*_POL_), and charge transfer between two monomers (Δ*E*_CT_):

4 $$\Delta E_{\mathrm{INT}}=\Delta E_{\mathrm{FRZ}}+\Delta E_{\mathrm{POL}}+\Delta E_{\mathrm{CT}}$$

The ALMO-EDA can also be performed self-consistently in the presence of implicit solvation (ALMO-EDA-PCM) where each term will consistently include the contribution from the implicit solvent.^62^ The frozen interaction Δ*E*_FRZ_ can also be further broken down into electrostatic interaction (Δ*E*_ELEC_), Pauli exclusion (Δ*E*_PAULI_) and dispersion (Δ*E*_DISP_) interactions.^87,88^ We note that Δ*E*_INT_ is not identical to Δ*E*_FORM_ because the former is computed from two fragments of an optimized dimer, whereas the latter optimizes the geometries of monomers and (H_2_O)_*n*_ independently.

The EDA analysis on the three dimer models of Table 1 is shown in Figure 4. Δ*E*_INT_ of 125.4 kJ/mol confirms that in the gas phase, IAHB dimer formation is disfavored (Figure 4a) i.e it is a metastable structure. The PCM solvent changes the formation energy by ∼200 kJ/mol, making dimer formation favorable. This change is driven by PCM-induced changes in the electrostatics, as is evident in the lower panel of Figure 4a. The −2 charge of the dimer induces a nearly opposite cavity surface counter-charge, which is primarily responsible for this shift. In addition, the explicit inclusion of the first solvation shell further stabilizes dimer formation by ∼45 kJ/mol. Figure 4a indicates that the net effect of water molecules in the first solvation shell is to stabilize the electrostatic component of Δ*E*_FRZ_. A revealing result of this analysis is that the large favorable interaction of two permanent electrostatics is offset by equally large, unfavorable steric hindrance highlighted by the large Pauli interaction (Figure 4a). Adding dispersion does not make Δ*E*_FRZ_ negative in any case. This highlights that while permanent electrostatic interaction is substantial as many have found before,^13,15,74^ it alone is insufficient to form the IAHB dimer. In other words, we show that describing the formation of the H_2_PO4^–^ dimer as the sole result of permanent electrostatics misses an important detail.

**Figure 4:**
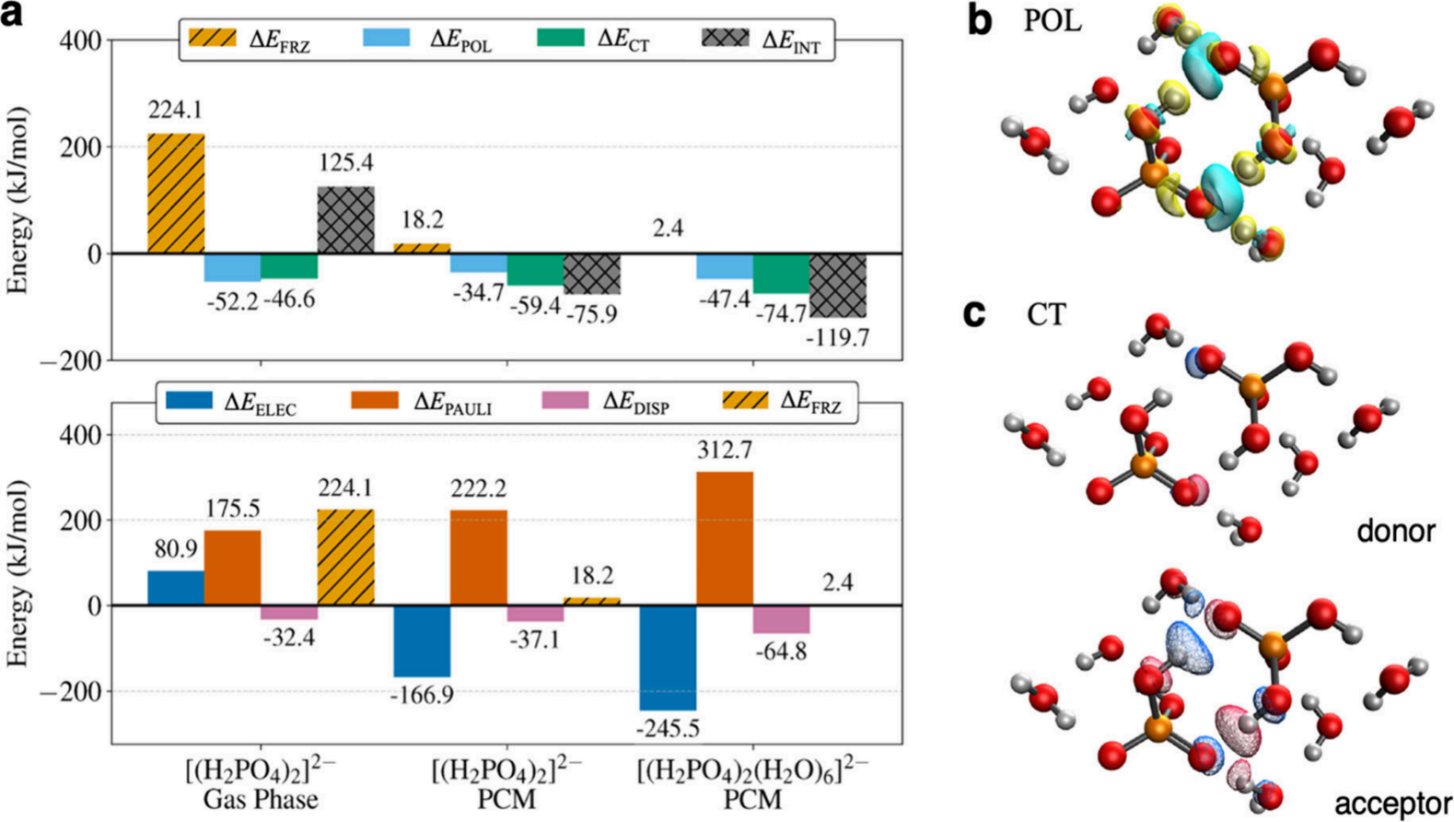
Energy decomposition analysis (EDA). (a) EDA results of three dimer systems with differing levels of solvation modeling. The top panel shows the decomposition of the entire interaction energy (Δ*E*_INΤ_) via eq 4, and the bottom panel shows the decomposition of the frozen interaction (Δ*E*_FRZ_). (b) Computed electronic density difference between states before and after polarization. Positive change in density is plotted in cyan and negative change in density is plotted in yellow. (c) Most significant complementary occupied/virtual pair (COVP)^65^ of the charge transfer step. The acceptor orbital is plotted with a meshed surface, and the donor orbital is plotted as a solid surface. The isosurface contains 35% electron density.

Figure 4a further shows that the formation of the dianionic PA dimer becomes favorable only after the addition of polarization and charge-transfer. Figure 4b,c shows that water molecules in the first solvation shell play a direct role in redistributing the electron density between the two fragments. The electronic density difference (EDD) between the two fragments before and after the orbital polarization (without any charge transfer) is shown in Figure 4b. The observed EDD extends to two adjacent waters that directly interact with the acceptor oxygen of H-bonds formed between the two PA fragments. No significant density change is identified in other waters, demonstrating that these two serve a particular role in responding to the frozen fragments. Indeed, analysis of the complementary occupied/virtual pair (COVP) orbitals^80^ formed during the charge transfer reveals that these waters also accept a portion of the electron density. The COVP shown in Figure 4c is the most significant donor–acceptor pair involved in charge transfer. The acceptor orbital (meshed surface) extends into the adjacent water molecules, demonstrating that the charge from the donor’s p-orbital, aligned along the hydrogen bond axis, is redistributed to these waters. Thus, we show that the solvent molecules do more than screening the locally accumulated charge. We show that in fact screening alone is not enough to form the IAHB dimer as it will be negated by steric hindrance. Instead, the fluctuation and reorganization of the charges that permeate outside two anion fragments allow the dimer to form the IAHB dimer. Figure 5 graphically summarizes the ALMO-EDA calculations where the electrostatic repulsion between two phosphates is compensated by solvation, polarization, and charge transfer, resulting in a favorable formation energy of a IAHB dimer.

**Figure 5:**
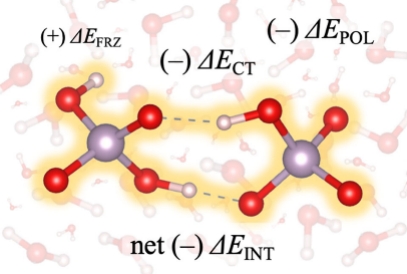
A graphical schematic of energy decomposition analysis (EDA) for phosphate IAHB dimer formation in a solvation environment. The net fragment interaction energy (Δ*E*_INT_) can be expressed as three energy terms accounting for frozen interactions (Δ*E*_FRZ_), polarization (Δ*E*_POL_), and H-bond charge transfer (Δ*E*_CT_). Their font sizes represent the relative amounts of favorable (−) and unfavorable (+) contributions to dimer formation, as quantified in Figure 4a.

## Conclusion

In conclusion, our integrated experimental and computational investigation provides compelling evidence for the formation of interanionic phosphate dimers in aqueous PA solutions, stabilized via solvation, polarization relaxation, and cyclic H-bonding. EXAFS data, supported by EA-TDDFT and EDA calculations, reveal the emergence of cyclic H-bonded structures at [PA] ≥ 7 M. NEXAFS further delineates the concentration-dependent disruption of the water H-bond network and the transition into more robust H-bonded interactions. These XAFS measurements offer structural evidence for IAHB dimer formation in solution, an interaction previously hypothesized but not experimentally verified in the absence of supramolecular anion acceptors. The data also offer critical structural and spectroscopic insight into the formation of extended H-bonded PA assemblies that support Grotthuss-type proton transport, consistent with the anomalously high conductivity observed in concentrated PA solutions.^29^

These findings challenge traditional electrostatic expectations and emphasize the role of water-mediated stabilization in enabling anion–anion association. Future studies employing time-resolved structural techniques, such as X-ray free-electron lasers and spectroscopic methods,^36^ will be vital to elucidate the dynamics of long-range hydrogen-bond networks in dense ionic environments (e.g., water-in-salt electrolytes and cytoplasm of halophilic microorganisms). Our combined experimental and theoretical strategy for probing interionic H-bonding can be extended to a wide range of ionic environments. For example, in an ionic liquid, tetracyanoborate anions have been shown to form dimers in both liquid and solid phases.^89^ Consistent with the theoretical framework presented here, stabilization by induction and dispersion between cyano groups can overcome electrostatic repulsion. These insights open opportunities to tune such interactions by varying counterions or functional groups and to interrogate the resulting structures using element-specific X-ray absorption at different edges. Such investigations may reveal broader implications for ion transport, the synthesis of tunable porous materials, and collective dynamics relevant to energy storage and biological systems.

## Supplementary Material





## Notes

Experimental methods, computational details, velocity-map image of photoelectrons, EXAFS data processing, contribution of scattering paths to EXAFS modeling, computed P–O radial density function, all EXAFS model fitting results, individual scattering paths shown in 12 M EXAFS, calculated natural transition orbitals for O 1s transitions, linear combination analysis of PA NEXAFS, all values of structural parameters obtained from the best fit to the EXAFS data (Tables S1–S3), and references (PDF)
Optimized geometries of the phosphoric acid dimer (ZIP)
