# Supplementary material for: Structural Evidence of Interanionic Hydrogen Bonding in Phosphoric Acid Solutions
Source: J Am Chem Soc. 2025 Nov 24;147(49):44916–25. doi: 10.1021/jacs.5c12699 (PMC12703727; doi:10.1021/jacs.5c12699)
Supplement: Supplementary file 1 [file ja5c12699_si_001.pdf]

Supporting Information for

Structural Evidence of Inter-Anionic Hydrogen Bonding in Phosphoric Acid Solutions

Pyeongeun Kim[1†], Richard Kang[1,2†], Kevin Carter-Fenk[1,2,3], Kevin R. Wilson[1], Martin Head-Gordon[1,2]*, and Musahid Ahmed[1]*

[1]Chemical Sciences Division, Lawrence Berkeley National Laboratory, Berkeley, CA 94720, USA

[2]Department of Chemistry, University of California, Berkeley, CA 94720, USA

[3]Department of Chemistry, University of Pittsburgh, Pittsburgh, PA 15218, USA

[†]P.K. and R.K. contributed equally to this work.

*Correspondence to m_headgordon@berkeley.edu and mahmed@lbl.gov

Table of Contents:

1. Experimental Methods ………………..…………..…………………...…… S2
2. Computational Details ………………………………………………….. S3
3. Velocity-map image of X-ray photoelectrons ………………………………... S5
4. EXAFS data processing …………………………………………………… S6
5. Contribution of scattering paths to EXAFS ………………………………….. S7
6. Computed P-O radial density function ………………………………………. S8
7. All EXAFS (FT) model fitting results ……………………………………….. S9
8. Contribution of scattering paths to EXAFS modeling (R-space)…………..… S10
9. Individual scattering paths shown in 10 M EXAFS ………………………… S11
10. Calculated natural transition orbitals for O 1s to σ and σ* transitions …….. S12
11. Linear combination analysis of PA NEXAFS spectra ……………………….. S13
12. Structural parameters obtained from the best fit to the EXAFS data ……… S14
13. References ………………………………………………………………… S17

Experimental Methods

X-ray absorption fine structure (XAFS) measurements

The O-K edge X-ray absorption fine structure data were collected using the Velocity Map Imaging photoelectron spectrometer at the Chemical Dynamics Beamline 9.0.1 (Advanced Light Source, Berkeley, California, USA). Details of the experimental layout is thoroughly discussed in previous papers from this group.[1–3] In brief, polydisperse phosphoric acid aerosols were generated by a constant output atomizer (TSI 3076) and guided into the X-ray interaction region by an aerodynamic lens and differential pumping. Similar to the liquid jet method, this aerosol-based technique enables radiation damage to the solution to be excluded. The picoammeter (Keithley 6485) connected to the photomultiplier tube (PMT, Hamamatsu) measured number of photoelectrons per second generated from the X-ray irradiation of phosphoric acid aerosols as a current (I_{PE}). The photoelectron signal is normalized with the current (I_{PD}) measured from the photodiode (PD) which measures the number of photons per second to yield the unitless intensity of X-ray absorption events ($I_{XAS} = I_{PE} / I_{PD}$). The optimal alignment of aerosol and X-ray beam was assured by ensuring the maximum intensity of secondary electrons viewed by photoelectron imaging CCD, which is switchable with PMT (Figure S1). Gas phase signals are subtracted by background measurements with HEPA-filtered aerosol flow. The range and interval of X-ray energies were adjusted for extended and near-edge XAFS (520 – 750 eV with ΔE = 1eV for EXAFS and 528 – 552 eV with ΔE = 0.2eV for NEXAFS). Solutions were prepared dissolving 85 wt% phosphoric acid solution (Alfa Aesar, 1:1 H_2O:H_3PO_4) in ultrapure water (Milli-Q system, 18MΩ/cm), to obtain 2 to 10 M concentration range. The PA concentration at the X-ray interaction region is ~20% higher than the solution, therefore, aerosol concentrations range from 2.4 to 12 M.

EXAFS Analysis

Normalization, reduction, and fitting of EXAFS data (2.4, 7.2, and 12 M) were performed with FEFF8 code and Larch software package.[4] Phases and amplitude function of each scattering path was calculated by the FEFF8 code using the Muffin-tin potential model (Figure S2). The fittings of molecular model to the k^2-weighted EXAFS spectra were made both in the k-space (1.8 – 7.2 Å^{-1}) and R-space (0.8 – 6 Å). The amplitude reduction factor (S_0^2) and change in edge energy (E_0) were set as global adjustable parameters while each scattering path has half scattering distance (R) and mean square relative displacement or Debye-Waller factor (σ^2) as fitting parameters. The degeneracy of each path (N) is set to constant according to the number of absorbing atoms in the model. Due to the relatively low degree of repeating structures in liquid sample, contrary to solid, different fitting schemes can be used to reproduce the data. The complete set of fitting schemes and parameters are tabulated in Table S1-3.

Because EXAFS analysis involves Fourier transformation of k-space data into R-space, real and imaginary R-space data is produced. Commonly for display and communication purposes, only the modulus of R-space data is shown. However, the modulus data does not effectively deliver the individual contribution of each scattering path. The modulus data is often the result of the

interference of complex scattering path terms. Therefore, to effectively show the contribution of shorter- and longer-range scattering paths, the absolute of real part of the scattering paths (|Re(χ(R))|) is overlaid with the modulus data in Figure 2 and Figure S4. In these figures, it can be observed that the |Re(χ(R))| is blanketed by the curve of the modulus, illustrating the actual contribution of scattering paths to the R-space EXAFS.

Computational Details

Two sets of classical *ab initio* molecular dynamics (AIMD) calculations were carried out using the CP2K package.[5] To sample the dimer and monomer configurations, the revised Perdew–Burke–Ernzerhof (RPBE) density functional[6] was used with DZVP-GTH[7] basis set on heavy atoms and SZVP-GTH[7] basis set on H atom in conjunction with the GTH pseudopotential.[8] A cubic simulation box with a side length of 16.003 Å was used, which contained 132 explicit waters for the monomer simulation and 127 waters for the dimer simulation. These conditions correspond to 0.405 M and 0.810 M H_3PO_4, respectively. For the dimer simulation, a classical MD simulation of 1 ns was first run to fix the P-P distance of the dimer at 3.8 Å. The MMFF force field was used as implemented in the Tinker-HP package.[9] The simulation was brought up to 300 K in 5 ps increments starting at 100 K for 5 ps, 200 K for 5 ps, and then 300 K for 500 ps. Using the final snapshot from classical MD, the AIMD simulation was run for 10 ps using a 0.5-fs timestep, heating the system up to 330 K via Nosé-Hoover thermostat within the NVT ensemble. [10] A 30K increase in the temperature (from 300K in classical MD to 330K in AIMD) was to accommodate the glassy dynamics observed that would prevent us from sampling sufficiently different liquid dimer configurations. Another 10 ps production run was then carried out, resulting in 41 structures in 0.25-ps intervals for XAS calculations.

Cluster geometries for XAS calculations (described below) were generated by taking the first two explicit solvation shells of H_3PO_4 monomer/dimer. In practice, this was done by considering only the explicit H_2O (or H_3O^+/OH^-) molecules that are within 5.14 Å radius from the center P-atom(s) of H_3PO_4 monomer and dimer. This cutoff was determined based on the P-O radial distribution function computed from the entire 20-ps AIMD trajectories (Figure S3).

All XAS calculations were performed with a development version of the Q-Chem 6.1 software package[11] using electron-affinity time-dependent density functional theory (EA-TDDFT)[12] under the Tamm-Dancoff approximation (TDA).[13] The rCAM-B3LYP density functional was employed.[14] A dense grid containing 99 radial points treated under the Euler-Maclaurin scheme[15] and 590 angular points using a Lebedev quadrature was used to evaluate the exchange-correlation potential.[16] To balance accuracy and computational cost, a mixed atomic orbital basis set was employed using aug-pcX-2[17] for oxygen atoms of phosphorus acid, pcSeg-1 for all H atoms, and aug-pcseg-1 for all solvent O atoms.[17] All XAS calculations employed a hybrid solvation model with the cluster snapshots containing two explicit water solvation shells around monomer and dimer embedded in a conductor-like polarizable continuum model (PCM)[18,19] with a dielectric constant of 78.39, using a solvent accessible surface (probe radius = 1.4 Å)[20] to capture the long-range electrostatics of the solution environment without allowing the dielectric medium to intercalate between explicit waters. The reported spectra were obtained by averaging 41 computed spectra from the trajectory snapshots, which were broadened with gaussians of standard deviation 0.15 eV.

The DFT analysis on the dimer formation also used the Q-Chem 6.1 software package.[11] To compute the dimer formation energy, each structure given in Eq. 1-3 was optimized individually with the ωB97M-V density functional[21] and def2-TZVPPD basis set[22] with PCM with a dielectric constant of 78.39. The energy decomposition analysis (ALMO-EDA) calculations were performed with the same DFT functional and basis set.

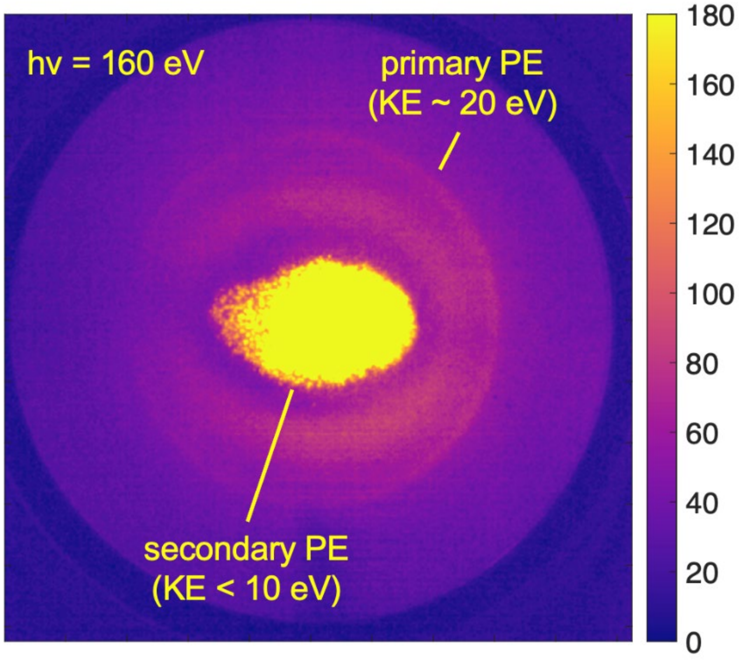

Figure S1. Velocity map image (VMI) of X-ray photoelectrons from aerosolized phosphoric acid (PA) solution captured during the optimization of aerosol beam–X-ray alignment. The incident X-ray energy was set to 160 eV to ionize the P 2p level (binding energy ≈ 140 eV) of aqueous PA, due to the brighter X-ray beam at this level compared to the beam energy at O 1s (>500 eV) used for main XAFS experiment. Primary photoelectrons (faint ring in the VMI) are emitted from the aerosol surface with a kinetic energy (KE) of ~20 eV, corresponding to the difference between photon energy and the P 2p binding energy. Secondary photoelectrons (bright spot at the center of VMI) originate from deeper within the droplets; after undergoing inelastic scattering, they emerge with reduced kinetic energy (KE < 10 eV). During XAFS measurements, the total intensity of VMI is captured by a PMT instead of the CCD camera.

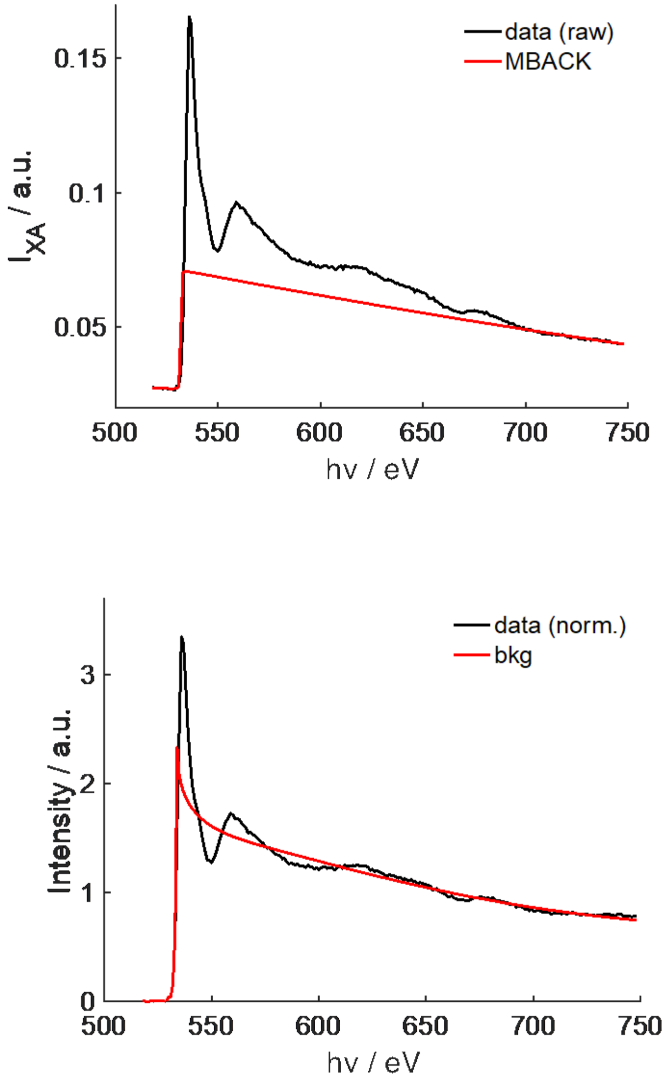

Figure S2. EXAFS data processing steps. (Top) Normalization of the 12 M PA spectra by using MBACK algorithm for photoelectron cross section. (Bottom) Background subtraction by using a spline function.

[PA] = 2.4 M

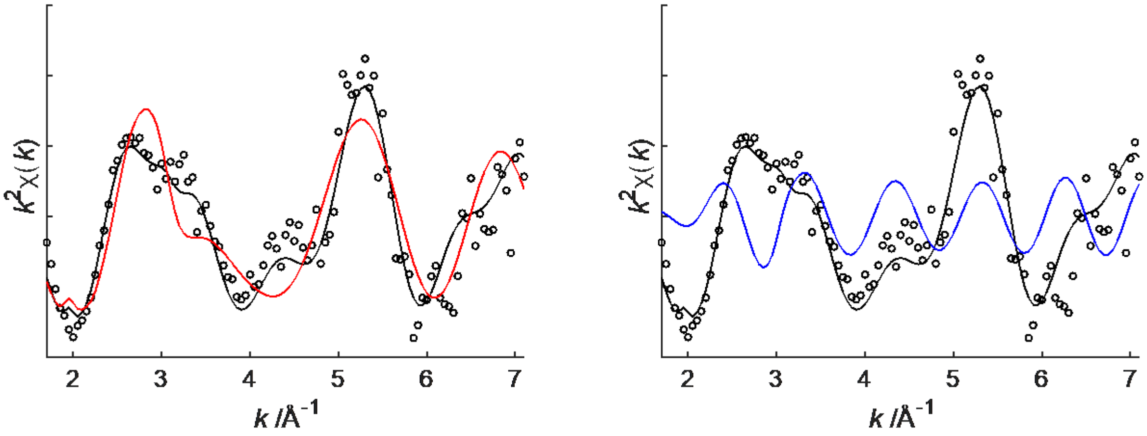

[PA] = 7.2 M

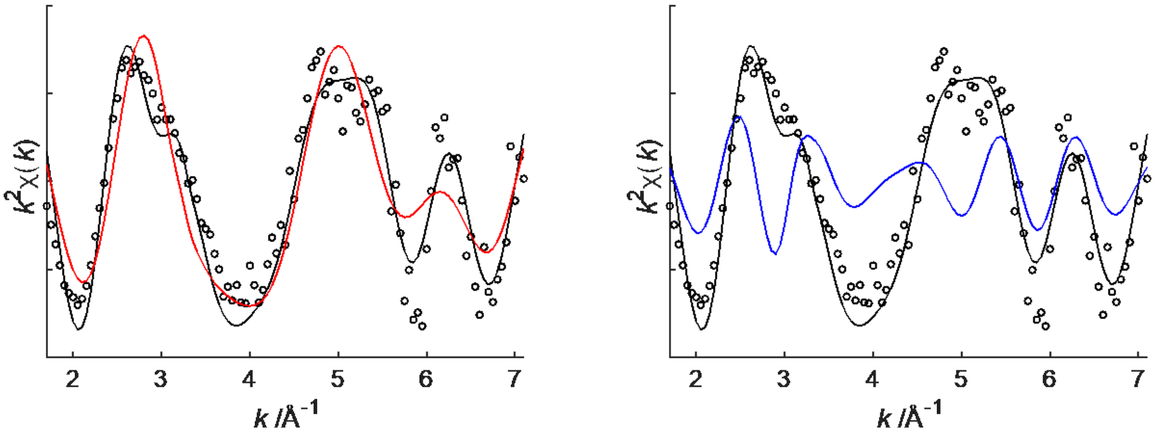

Figure S3. Contribution of short- and long-range scattering paths deconvoluted in EXAFS modeling (k-space). Top – 2.4 M and Bottom – 7.2 M.

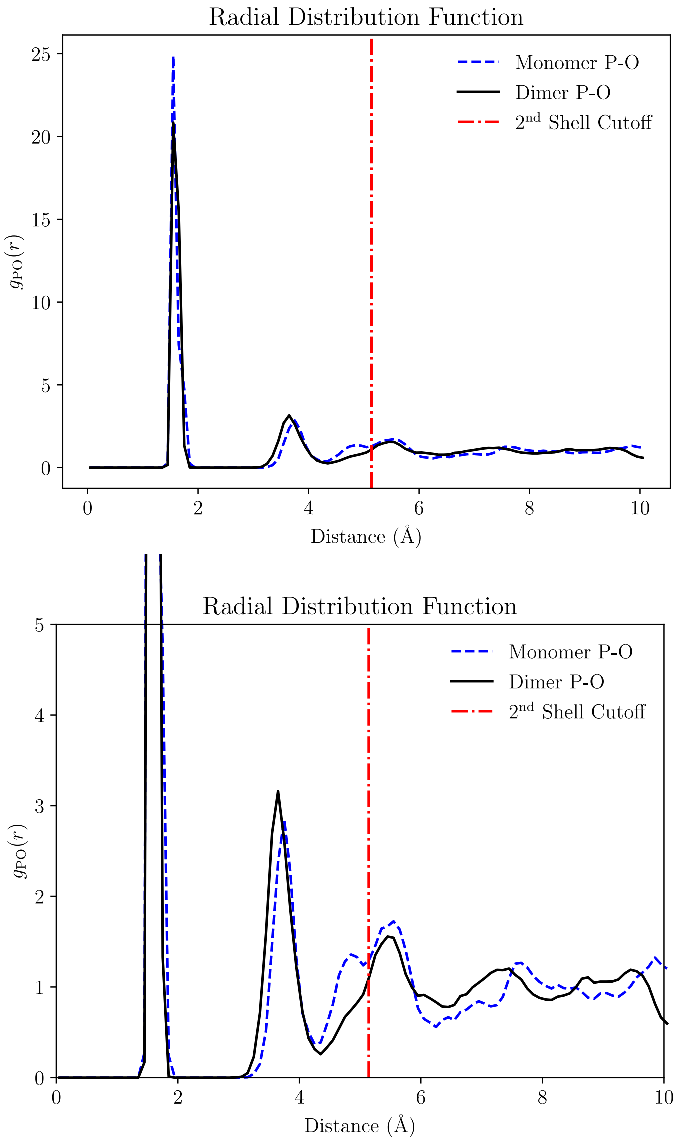

S

Figure S4. Computed P-O Radial Density Function computed from 20-ps AIMD trajectory. The first two solvation shell was determined by locating the first two peaks of non-bonded P-O distances. The first peak located at ~1.9 Å corresponds to the bonded P-O distances within monomer/dimer. The determined cutoff value used for second solvation shell cluster geometries was 5.14 Å. The bottom panel provides a magnified view of the data presented in the top panel.

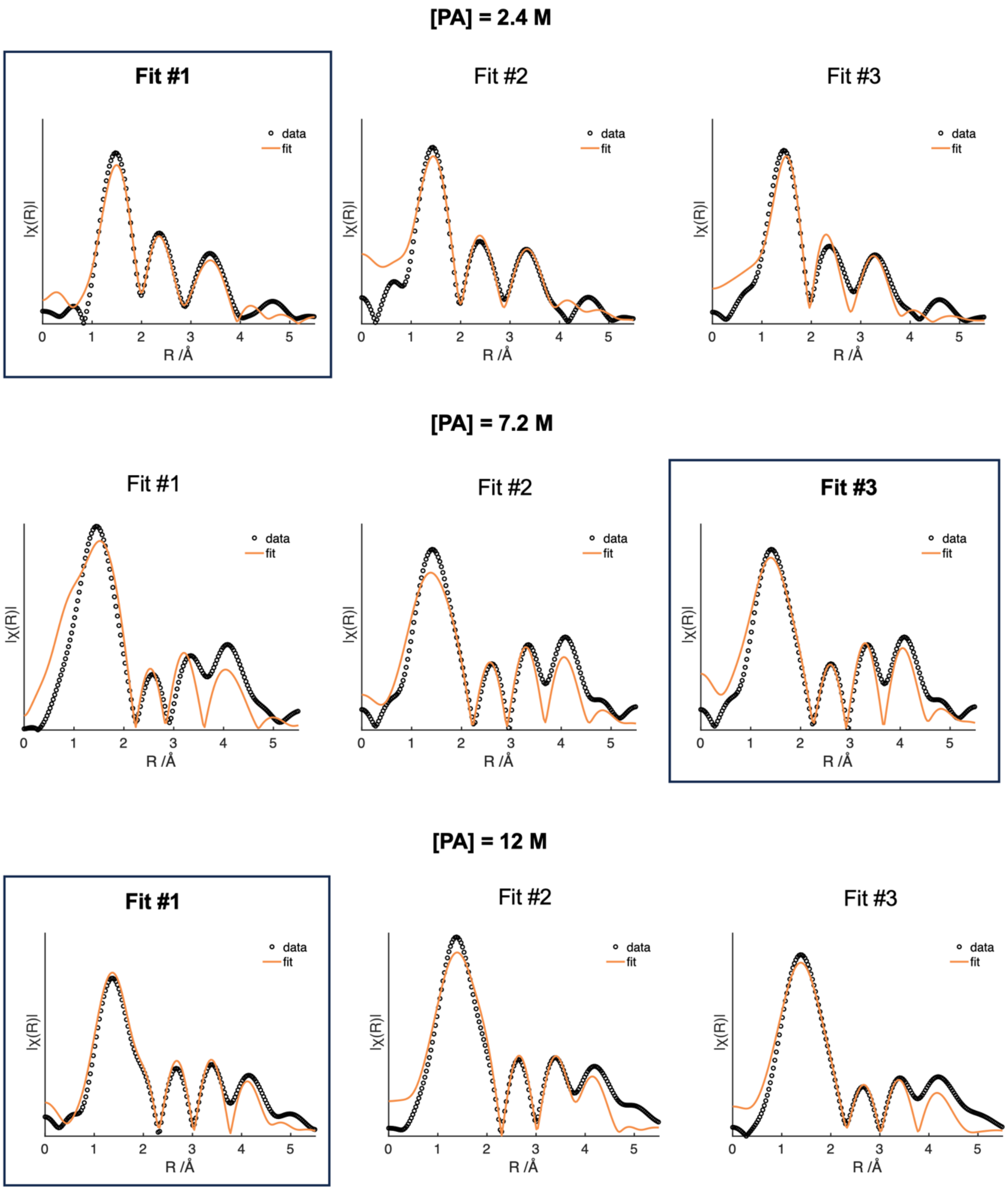

Figure S5. All model fitting result for measured EXAFS (Fourier transformed) of PA aerosols. Best fitting results used in the main text are highlighted with black boxes. Values of the fitting parameters are tabulated in Table S1–3.

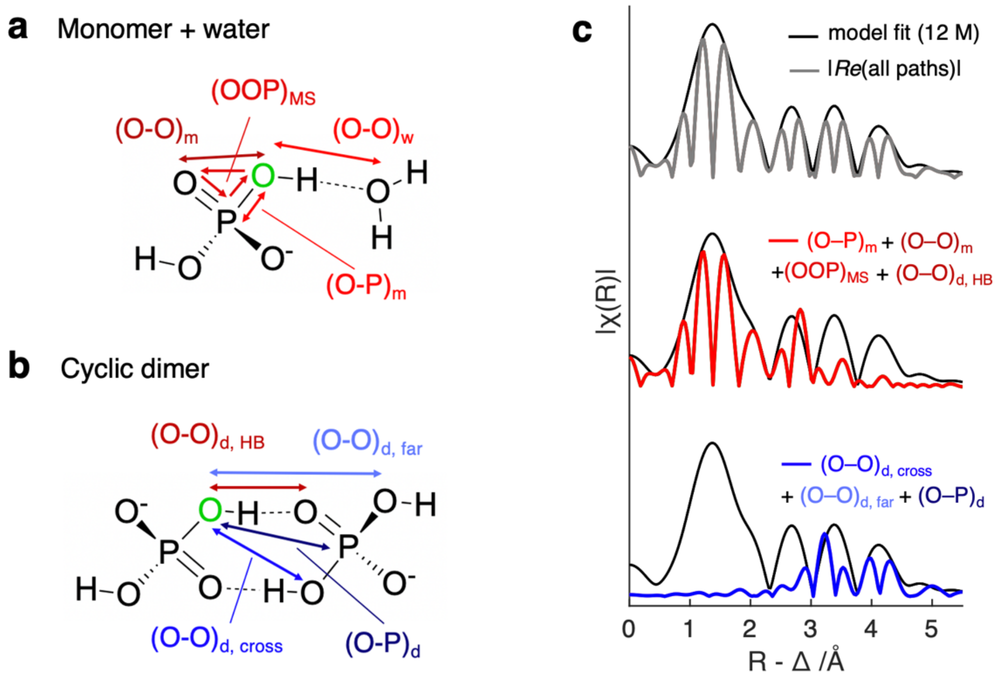

Figure S6. Contribution of short- and long-range scattering paths deconvoluted in EXAFS modeling of 12 M PA (R-space).

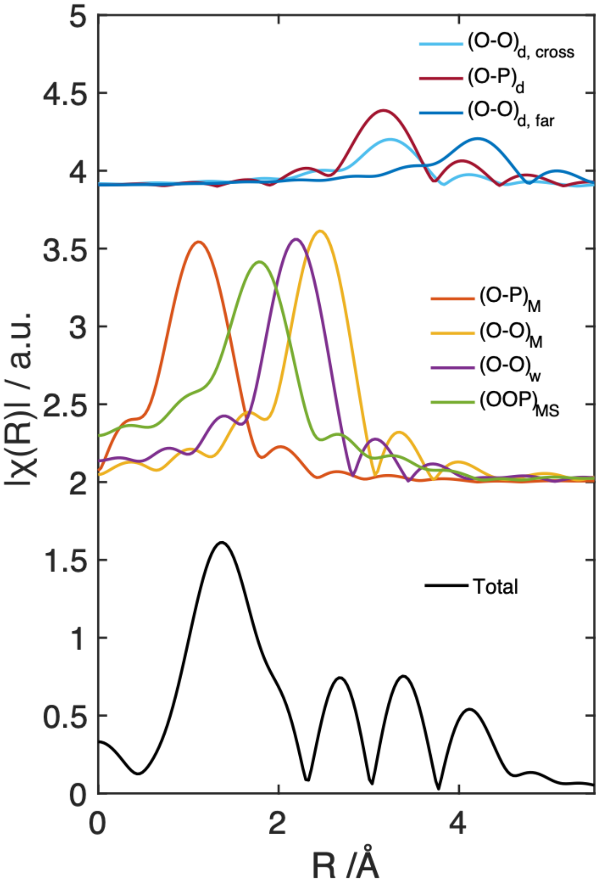

Figure S7. Longer-range (top) and shorter-range (middle) scattering paths of [PA] = 12 M shown individually along with total R-space EXAFS (bottom).

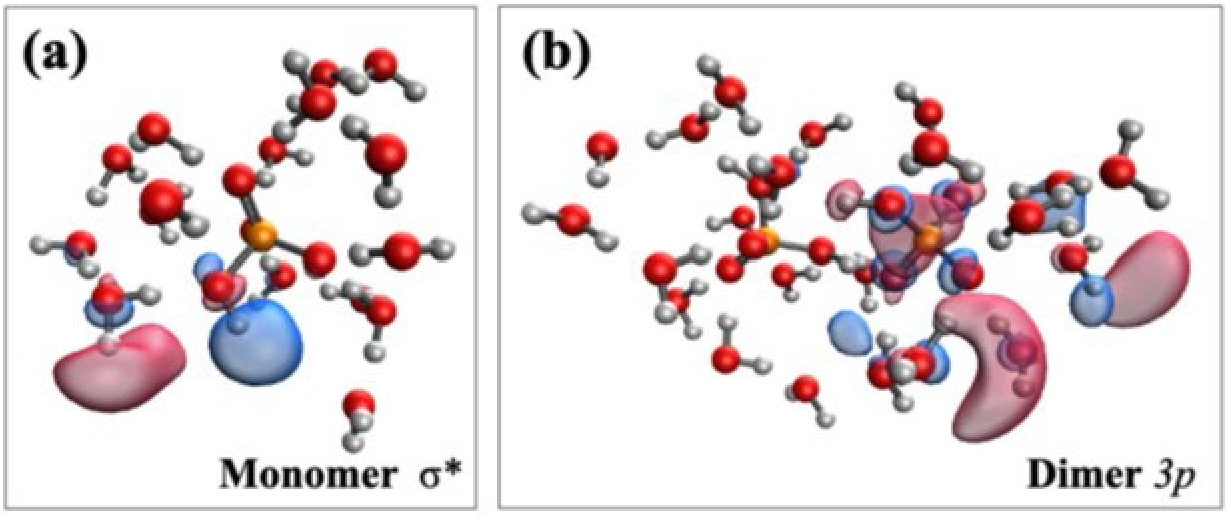

Figure S8. Calculated particle Natural Transition Orbitals[23] for O 1s excitations from PA. The orange, red, and white atoms indicate phosphorous, oxygen, and hydrogen atoms, respectively. All isosurfaces contain 30% of the total electron density. (a) The particle orbital for the monomer O 1s to OH σ* transition with partial charge transfer. (b) The particle orbital for the dimer O 1s to 3p transition.

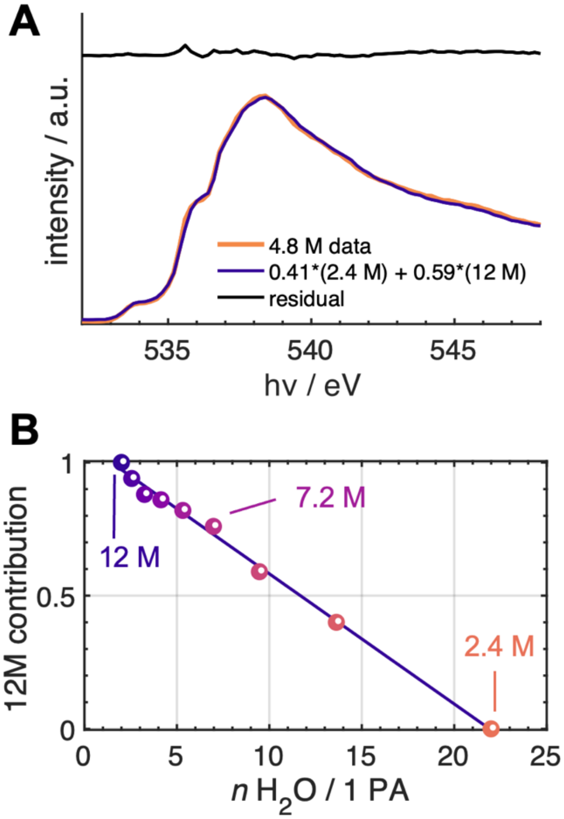

Figure S9. Linear combination analysis of PA NEXAFS spectra. (**A**) Decomposition of 4.8 M spectrum. (**B**) Linear relationship of dimer-like (12 M) contribution and H_2O:PA molar ratio. Representative molar concentrations of the solutions are indicated.

Table S1. Structural Parameters Obtained from the Best Fit to the EXAFS Data (2.4 M H_3PO_4). Unit for interatomic distance (*r*) is Å. The fitting parameters used in the main text for representation is noted with an asterisk (*).

Scattering path		Fit 1*	Fit 2	Fit 3
$(O–P)_m$	*N*	3.5	3.5	4
	r	1.58	1.58	1.58
	σ^2	0.040	0.035	0.046
$(O–O)_m$	*N*	11.5	11.5	12
	r	2.60	2.61	2.63
	σ^2	0.018	0.010	0.011
$(OOP)_{MS}$	*N*	12	12	12
	r	2.86	2.86	2.89
	σ^2	0.009	0.001	0.001
$(O–O)_w$	*N*	8	7	7
	r	2.75	2.77	2.77
	σ^2	0.036	0.014	0.019
$(O_w–P)$	*N*	4	6	7
	r	3.64	3.74	3.81
	σ^2	0.0021	0.0084	0.044
S_0^2		0.69	1.05	1.2
E_0 / eV		7.2	2.1	1.8
R-factor		0.028	0.020	0.054
N variables		12	12	14
N independent		14.06	14.06	18.88

Table S2. Structural Parameters Obtained from the Best Fit to the EXAFS Data (7.2 M H_3PO_4). Unit for interatomic distance (*r*) is Å. The fitting parameters used in the main text for representation is noted with an asterisk (*).

Scattering path		Fit 1	Fit 2	Fit 3*
$(O–P)_m$	*N*	4	4	3.5
	r	1.57	1.57	1.57
	σ^2	0.027	0.029	0.026
$(O–O)_m$	*N*	12	12	11.5
	r	2.61	2.54	2.54
	σ^2	0.024	0.05	0.03
$(OOP)_{MS}$	*N*	12	12	12
	r	2.85	2.86	2.85
	σ^2	0.038	0.020	0.018
$(O–O)_{d, HB}$	*N*	8	4	4
	r	2.70	2.66	2.66
	σ^2	0.021	0.005	0.001
$(O–O)_{d, cross}$	*N*	4	4	4
	r	3.48	3.48	3.48
	σ^2	0.001	0.001	0.001
$(O–P)_d$	*N*	4	4	4
	r	3.75	3.63	3.64
	σ^2	0.001	0.001	0.001
$(O–O)_{d, far}$	*N*	8	8	8
	r	4.87	4.72	4.72
	σ^2	0.001	0.0013	0.0013
S_0^2		0.6	0.6	0.7
E_0 / eV		4.2	3.9	3.6
R-factor		0.087	0.094	0.070
N variables		14	14	14
N independent		18.19	14.06	14.06

Table S3. Structural Parameters Obtained from the Best Fit to the EXAFS Data (12M H_3PO_4). Unit for interatomic distance (*r*) is Å. The fitting parameters used in the main text for representation is noted with an asterisk (*).

Scattering path		Fit 1*	Fit 2	Fit 3
$(O–P)_m$	*N*	3.5	4	3.5
	r	1.65	1.58	1.65
	σ^2	0.020	0.025	0.020
$(O–O)_m$	*N*	11	12	11
	r	2.60	2.60	2.60
	σ^2	0.001	0.001	0.0012
$(OOP)_{MS}$	*N*	12	-	12
	r	2.86	-	2.86
	σ^2	0.009	-	0.009
$(O–O)_{d, HB}$	*N*	8	4	8
	r	2.61	2.61	2.61
	σ^2	0.001	0.053	0.001
$(O–O)_{d, cross}$	*N*	4	4	4
	r	3.69	3.69	3.69
	σ^2	0.001	0.001	0.001
$(O–P)_d$	*N*	4	4	4
	r	3.72	3.72	3.72
	σ^2	0.001	0.001	0.001
$(O–O)_{d, far}$	*N*	8.5	8	8.5
	r	4.61	4.61	4.61
	σ^2	0.0013	0.0015	0.001
$(O–O)_w$	*N*	-	8	-
	r	-	2.54	-
	σ^2	-	0.0035	-
S_0^2		0.8	0.7	0.8
E_0		6.5	13.7	6.5
R-factor		0.044	0.034	0.050
N variables		13	16	14
N independent		14.06	14.57	17.55

References

(1) Kostko, O.; Jacobs, M. I.; Xu, B.; Wilson, K. R.; Ahmed, M. Velocity Map Imaging of Inelastic and Elastic Low Energy Electron Scattering in Organic Nanoparticles. *J. Chem. Phys.* **2019**, *151* (18), 184702.

(2) Weeraratna, C.; Kostko, O.; Ahmed, M. An Investigation of Aqueous Ammonium Nitrate Aerosols with Soft X-Ray Spectroscopy. *Mol. Phys.* **2022**, *120* (1–2), e1983058.

(3) Kim, P.; Weeraratna, C.; Nemšák, S.; Dias, N.; Lemmens, A. K.; Wilson, K. R.; Ahmed, M. Interfacial Nanostructure and Hydrogen Bond Networks of Choline Chloride and Glycerol Mixtures Probed with X-Ray and Vibrational Spectroscopies. *J. Phys. Chem. Lett.* **2024**, *15* (11), 3002–3010.

(4) Newville, M. Larch: An Analysis Package for XAFS and Related Spectroscopies. *J. Phys.: Conf. Ser.* **2013**, *430*, 012007.

(5) Kühne, T. D.; Iannuzzi, M.; Del Ben, M.; Rybkin, V. V.; Seewald, P.; Stein, F.; Laino, T.; Khaliullin, R. Z.; Schütt, O.; Schiffmann, F. CP2K: An Electronic Structure and Molecular Dynamics Software Package-Quickstep: Efficient and Accurate Electronic Structure Calculations. *J. Chem. Phys.* **2020**, *152* (19), 194103.

(6) Hammer, B.; Hansen, L. B.; Nørskov, J. K. Improved Adsorption Energetics within Density-Functional Theory Using Revised Perdew-Burke-Ernzerhof Functionals. *Phys. Rev. B* **1999**, *59* (11), 7413.

(7) VandeVondele, J.; Hutter, J. Gaussian basis sets for accurate calculations on molecular systems in gas and condensed phases. *J. Chem. Phys.* **2007**, *127* (11). 114105

(8) Goedecker, S.; Teter, M.; Hutter, J. Separable dual-space Gaussian pseudopotentials. *Phys. Rev. B* **1996,** *54* (3), 1703.

(9) Harger, M.; Li, D.; Wang, Z.; Dalby, K.; Lagardère, L.; Piquemal, J.-P.; Ponder, J.; Ren, P. Tinker-OpenMM: Absolute and Relative Alchemical Free Energies Using AMOEBA on GPUs. *J. Comput. Chem.* **2017**, *38* (23), 2047–2055.

(10) Nosé, S. A Unified Formulation of the Constant Temperature Molecular Dynamics Methods. *J. Chem. Phys.* **1984**, *81* (1), 511–519.

(11) Epifanovsky, E.; Gilbert, A. T.; Feng, X.; Lee, J.; Mao, Y.; Mardirossian, N.; Pokhilko, P.; White, A. F.; Coons, M. P.; Dempwolff, A. L. Software for the Frontiers of Quantum Chemistry: An Overview of Developments in the Q-Chem 5 Package. *J. Chem. Phys.* **2021**, *155* (8), 084801.

(12) Carter-Fenk, K.; Cunha, L. A.; Arias-Martinez, J. E.; Head-Gordon, M. Electron-Affinity Time-Dependent Density Functional Theory: Formalism and Applications to Core-Excited States. *J. Phys. Chem . Lett.* **2022**, *13* (41), 9664–9672.

(13) Hirata, S.; Head-Gordon, M. Time-Dependent Density Functional Theory for Radicals: An Improved Description of Excited States with Substantial Double Excitation Character. *Chem. Phys. Lett.* **1999**, *302* (5–6), 375–382.

(14) Cohen, A. J.; Mori-Sánchez, P.; Yang, W. Development of Exchange-Correlation Functionals with Minimal Many-Electron Self-Interaction Error. *J. Chem. Phys.* **2007**, *126* (19), 191109.

(15) Murray, C. W.; Handy, N. C.; Laming, G. J. Quadrature Schemes for Integrals of Density Functional Theory. *Mol. Phys.* **1993**, *78* (4), 997–1014.

(16) Lebedev, V. I. Values of the Nodes and Weights of Quadrature Formulas of Gauss–Markov Type for a Sphere from the Ninth to Seventeenth Order of Accuracy That Are Invariant with Respect to an Octahedron Group with Inversion. *Zhurnal Vychislitel'noi Matematiki i Matematicheskoi Fiziki* **1975**, *15* (1), 48–54.

(17) Ambroise, M. A.; Dreuw, A.; Jensen, F. Probing Basis Set Requirements for Calculating Core Ionization and Core Excitation Spectra Using Correlated Wave Function Methods. *J. Chem. Theory Comput.* **2021**, *17* (5), 2832–2842.

(18) Barone, V.; Cossi, M.; Tomasi, J. Geometry Optimization of Molecular Structures in Solution by the Polarizable Continuum Model. *J. Comput. Chem.* **1998**, *19* (4), 404–417.

(19) Truong, T. N.; Stefanovich, E. V. A New Method for Incorporating Solvent Effect into the Classical, Ab Initio Molecular Orbital and Density Functional Theory Frameworks for Arbitrary Shape Cavity. *Chem. Phys. Lett.* **1995**, *240* (4), 253–260.

(20) Herbert, J. M. Dielectric Continuum Methods for Quantum Chemistry. *Comput. Mol. Sci.* **2021**, *11* (4), e1519.

(21) Mardirossian, N.; Head-Gordon, M. ωB97M-V: A Combinatorially Optimized, Range-Separated Hybrid, Meta-GGA Density Functional with VV10 Nonlocal Correlation. *J. Chem. Phys.* **2016**, *144* (21), 214110.

(22) Weigend, F.; Ahlrichs, R. Balanced Basis Sets of Split Valence, Triple Zeta Valence and Quadruple Zeta Valence Quality for H to Rn: Design and Assessment of Accuracy. *Phys. Chem. Chen. Phys.* **2005**, *7* (18), 3297–3305.

(23) Martin, R. L. Natural Transition Orbitals. *J. Chem. Phys.* **2003**, *118* (11), 4775–4777.
